# What Shapes Quality of Life in Youth? A Multidimensional Approach from Lifestyle to Residential Context—Cor-School Study

**DOI:** 10.3390/healthcare13111256

**Published:** 2025-05-26

**Authors:** Alvaro Pano-Rodriguez, Saül Aixa-Requena, Jose Vicente Beltran-Garrido, Abraham Batalla-Gavaldà, Vicenç Hernández-González, Enric Conesa-Milian, Isaac López-Laval, Joaquin Reverter-Masia

**Affiliations:** 1 Faculty of Education, Psychology and Social Work, Department of Specific Didactics, University of Lleida, 25001 Lleida, Spain; saul.aixa@udl.cat (S.A.-R.); vicenc.hernandez@udl.cat (V.H.-G.); enric.conesa@udl.cat (E.C.-M.); joaquim.reverter@udl.cat (J.R.-M.); 2 Human Movement Research Group (RGHM), University of Lleida, Plaça de Víctor Siurana, 25003 Lleida, Spain; 3 Physical Exercise and Performance Research Group, Universidad Cardenal Herrera-CEU, CEU Universities, 12006 Castellon de la Plana, Spain; 4 Department of Education Science, School of Humanities and Communication Sciences, Universidad Cardenal Herrera-CEU, CEU Universities, 12006 Castellon de la Plana, Spain; 5 University School of Health and Sport (EUSES), Universitat Rovira i Virgili, 43870 Amposta, Spain; a.batalla@euseste.es; 6 Department of Education and Specific Didactics, Faculty of Humanities and Social Sciences, Universitat Jaume I, 12071 Castellon de la Plana, Spain; 7 Faculty of Health and Sport Science, Department of Physiatry and Nursing, University of Zaragoza, 22071 Huesca, Spain; isaac@unizar.es

**Keywords:** health-related quality of life, children and adolescents, physical activity, Mediterranean diet, sleep quality, urban–rural differences, cardiometabolic health

## Abstract

**Background**: Health-related quality of life is a key indicator of well-being that integrates physical, emotional, and social dimensions. Identifying its determinants during childhood and adolescence is essential to guide effective health promotion strategies. **Objective**: To analyze the association between health-related quality of life and demographic, anthropometric, biochemical, lifestyle, and environmental variables in school-aged children and adolescents. **Methods**: A cross-sectional study was conducted during the 2023–2024 academic year in northeastern Spain. A total of 571 children and adolescents participated in the study (mean age = 11.64 ± 1.64 years), of whom 254 were girls (44.48%). Health-related quality of life was assessed using the Revised Children’s Quality of Life Questionnaire (KINDL-R), a validated instrument for assessing perceived well-being in pediatric populations. Independent variables included waist-to-height ratio, high-density lipoprotein cholesterol, low-density lipoprotein cholesterol, adherence to the Mediterranean diet, weekly physical activity, sleep quality, and residential context (i.e., large urban areas, small urban areas, and municipalities). Multiple linear regression analysis was performed to explore the associations. **Results**: Older age was associated with lower health-related quality of life (β = −1.07; 95% CI: −1.61 to −0.53; *p* < 0.001). In contrast, better adherence to the Mediterranean diet (β = 0.72; 95% CI: 0.09 to 1.37; *p* = 0.023), better sleep quality (b = −2.48; *p* < 0.001), and participants living in cities and large urban areas reported compared to those in municipalities were associated with higher HRQoL (*p* < 0.05), with moderate effect sizes. No significant associations were found for sex, waist-to-height ratio, cholesterol levels, or physical activity. **Conclusions**: This study highlights the importance of adopting a multidimensional approach to promote health-related quality of life in young populations, addressing dietary habits, sleep quality, and environmental factors within health promotion strategies.

## 1. Introduction

Health-related quality of life (HRQoL) is increasingly recognized as a key indicator of overall health and well-being, particularly in children and adolescents, where it encompasses physical, emotional, social, and school-related dimensions [1,2]. HRQoL is a multidimensional construct encompassing physical, emotional, social, and school functioning. It reflects the individual’s subjective perception of well-being across these domains and is widely used in pediatric research as an outcome indicator of health and developmental status [3]. The assessment of HRQoL during these life stages provides valuable information not only about current health status but also about potential trajectories of health and disease into adulthood [4,5]. Understanding the factors that influence HRQoL in youth is therefore essential for the development of effective preventive and health promotion strategies.

Traditionally, research has identified several individual determinants of HRQoL in pediatric populations, including sex and age [6]. Girls frequently report lower HRQoL scores than boys, particularly in psychological domains [7], while older adolescents tend to experience a progressive decline in HRQoL, possibly related to increasing academic demands, body image concerns, or psychosocial stressors [8].

Beyond sociodemographic characteristics, growing attention has been paid to modifiable lifestyle factors, such as diet, physical activity, and sleep quality, given their established relevance to both physical and mental health [9,10,11,12]. In this regard, adherence to the Mediterranean diet, characterized by high consumption of fruits, vegetables, whole grains, legumes, and olive oil, has shown a positive association with HRQoL in children and adolescents [12]. Similarly, higher levels of physical activity have been consistently linked to improved HRQoL, particularly in terms of physical well-being, vitality, and emotional regulation [13]. Sleep quality has also emerged as a critical, yet sometimes overlooked, determinant of HRQoL, with evidence indicating that insufficient or poor-quality sleep can negatively affect mood, cognitive performance, and social relationships in youth [14,15].

Anthropometric indicators, particularly those reflecting body composition, have also been associated with HRQoL. While most studies have focused on body mass index as a general measure of weight status, other indicators such as the waist-to-height ratio (WtH) may provide additional information regarding central adiposity and cardiometabolic risk [16,17]. Central fat accumulation has been independently linked to reduced HRQoL in pediatric populations, potentially due to its associations with self-perception, body image, and health-related limitations [18].

Biochemical markers, such as lipid profile parameters (HDL and LDL cholesterol), have received comparatively less attention in relation to HRQoL in youth. However, dyslipidemia in children has been related not only to future cardiovascular risk but also to unhealthy lifestyle patterns, such as poor diet or low physical activity, which may indirectly influence HRQoL [19]. Understanding the role of these biohealth indicators could therefore offer a more comprehensive view of the factors underpinning subjective well-being in this population.

Furthermore, the living environment represents another potentially relevant determinant of HRQoL. The residential context encompasses a range of environmental characteristics that may influence children’s access to health-promoting resources such as green spaces, recreational facilities, and social support networks. Differences in infrastructure, service availability, and population density between urban, semi-urban, and rural areas may shape the conditions for physical and psychological well-being. In this study, the residential context is operationalized using a population-based classification (urban, semi-urban, rural), as defined by the Spanish Atlas of Urban Areas [20], and is analyzed in relation to self-perceived quality of life. While some studies suggest that residing in rural or small-town environments may foster better HRQoL due to greater social cohesion and contact with nature [21], other evidence indicates that urban areas may provide better access to health resources, sports facilities, or leisure activities, which could enhance HRQoL [22]. However, the findings are heterogeneous and may vary depending on the specific context and population analyzed. For instance, a study conducted in Spain found that adolescents residing in rural areas reported higher HRQoL scores, particularly in psychological well-being and school environment dimensions, compared to their urban counterparts [23]. Conversely, research from the Czech Republic indicated that urban residents reported higher overall quality of life, suggesting that the relationship between urbanization and HRQoL is influenced by regional and cultural factors [24].

Despite the growing body of research addressing the determinants of HRQoL in youth, most previous studies have focused on isolated variables, providing a fragmented view of the phenomenon. There is a clear need for studies adopting a multifactorial and integrative approach that simultaneously considers behavioral, anthropometric, biochemical, and environmental factors in the same analytical model. This multidimensional approach is essential to understanding HRQoL in childhood, as biological, behavioral, and environmental factors do not operate in isolation. For example, unhealthy behaviors such as poor diet or insufficient physical activity may contribute to unfavorable anthropometric or metabolic profiles, which in turn can affect subjective well-being. Simultaneously, environmental factors such as urban infrastructure or access to green spaces may facilitate or hinder healthy habits, creating reinforcing patterns that influence both health outcomes and quality of life perceptions. Considering these interconnections is crucial to fully capture the complexity of pediatric HRQoL and to design effective interventions.

In this context, the present study aims to fill this gap by analyzing the combined and independent associations between HRQoL, and a comprehensive set of variables, including sex, age, WtH, lipid profile (HDL and LDL), adherence to the Mediterranean diet, physical activity levels, sleep quality, and type of population (urban vs. rural) in a large and diverse sample of school-aged children and adolescents. By adopting this multifactorial perspective, our study seeks to provide new insights into the complex interplay of biological, behavioral, and environmental factors influencing HRQoL, with relevant implications for the design of integrated health promotion strategies in pediatric settings.

## 2. Materials and Methods

### 2.1. Study Design

This study employed a cross-sectional design and was conducted in the provinces of northeastern Spain. The research was part of the Cor-School Study, a broader project aimed at analyzing lifestyle-related cardiometabolic health indicators in the school-aged population.

A stratified random sampling strategy was applied to ensure a diverse representation of extracurricular sports programs across different geographical areas (urban vs. rural settings). This approach sought to capture potential environmental and contextual differences that might influence HRQoL, health-related behaviors, and outcomes. Data collection was conducted between October 2023 and February 2024, corresponding to the first and second terms of the academic year.

The study protocol was developed in accordance with the ethical principles of the Declaration of Helsinki [25] and was approved by the Ethics Committee for Clinical Research of the Catalan Council (30/CEICGC/2020).

### 2.2. Participants

A total of 571 schoolchildren participated in this study, including 254 girls and 358 boys, with a mean age of 11.64 years (SD = 1.64), ranging in age from 8 to 16 years. Participants were contacted through their schools and extracurricular sports programs, which served as intermediaries in distributing the study invitation and information sheets. Schools and programs that agreed to collaborate defined the sampling frame. Within these, all students meeting the inclusion criteria were invited to participate. Inclusion criteria comprised being enrolled in primary education, providing informed consent signed by parents or legal guardians, and the ability to complete the self-reported fitness questionnaire. Exclusion criteria included the presence of any medical condition that could significantly influence physical activity behaviors, as reported by the participants themselves.

All parents or legal guardians provided written informed consent prior to participation. In addition, they received detailed information regarding the objectives and procedures of the study, as well as any potential risks or inconveniences derived from participation, which were minimal given the observational and non-invasive nature of the study. Participation was entirely voluntary, and confidentiality and data protection were guaranteed in accordance with current ethical standards and applicable regulations.

A statistical power assessment was performed using G*Power3 (v. 3.1.9.6 for Mac) [26] to estimate the minimum detectable effect size (*f*^2^) for a multivariate regression analysis. With a sample size of 571 participants, 9 predictor variables, a significance threshold of α = 0.05, and 80% statistical power (1 − β = 0.80), the analysis demonstrated that the model could consistently identify effects as low as *f*^2^ = 0.02. This value aligns with a minimal effect size under Cohen’s established criteria (*f*^2^ ≥ 0.02, minimal; ≥0.15, moderate; ≥0.35, substantial), indicating sufficient analytical precision to detect weak associations within the target population.

### 2.3. Instruments and Variables

#### 2.3.1. Quality of Life

HRQoL was assessed using the KINDL-R questionnaire, a validated instrument designed to evaluate HRQoL in children and adolescents aged 3 to 17 years [27]. In this study, the self-report version for children aged 8 to 16 years was used. This version is appropriate for children who are able to read and understand the items independently and has demonstrated good internal consistency, with Cronbach’s alpha coefficients exceeding 0.75 in all subscales and reaching 0.95 for the total scale. Its validity has been supported through significant correlations with other established instruments such as the Short Form Health Survey (SF-36) and the Questions on Life Satisfaction Module (FLZ), and it has shown sensitivity to change and good acceptability among children aged 8 to 16 years, with high completion rates and minimal missing data [1]. The instrument includes 24 items covering six dimensions: physical well-being, emotional well-being, self-esteem, family, friends, and school. Each item is rated on a 5-point Likert scale, and scores are transformed to a 0–100 scale, with higher scores indicating better perceived quality of life.

Participants completed the questionnaire individually, under the supervision of trained researchers who provided clarifications when necessary.

#### 2.3.2. Anthropometric Variables

Anthropometric assessments were performed in accordance with the protocols established by the International Society for the Advancement of Kinanthropometry (ISAK) [28]. Body weight was assessed with a Seca 877 digital scale (Hamburg, Germany) accurate to ±0.1 kg, and stature was measured using a Seca 213 portable stadiometer (Hamburg, Germany) with a precision of ±0.1 cm. Waist-to-height ratio (WtH) was subsequently calculated as waist circumference by height (cm/cm).

Skinfold thickness measurements were obtained at standard anatomical landmarks using a calibrated skinfold caliper (Harpenden, Baty International, Hertfordshire, UK; precision ±0.2 mm). In addition, a flexible, non-elastic tape (Lufkin W606PM, Apex Tool Group, York, PA, USA) was employed to measure body girths as required.

Fat mass and muscle mass percentages were estimated using specific equations validated for the pediatric population and recommended by ISAK for this type of assessment [28].

#### 2.3.3. Biochemical Variables

Fasting blood samples were collected by qualified nursing staff to assess lipid profile parameters. Specifically, high-density lipoprotein cholesterol (HDL-C) and low-density lipoprotein cholesterol (LDL-C) concentrations were determined using enzymatic colorimetric methods in accredited laboratories [29].

These biochemical markers were included due to their recognized relevance in cardiovascular risk assessment and their potential associations with lifestyle behaviors in children and adolescents [30].

#### 2.3.4. Lifestyle Variables

Adherence to the Mediterranean Diet: Adherence to the Mediterranean diet was assessed using the KIDMED index [31], which evaluates dietary patterns based on the consumption frequency of foods characteristic of the Mediterranean dietary model. The index provides scores ranging from 0 to 12, with higher scores indicating better adherence.

Physical Activity Levels: Physical activity levels were assessed using the International Physical Activity Questionnaire for Children (IPAQ-C) [32], which estimates the total amount of moderate-to-vigorous physical activity performed during a typical week. Results were expressed as MET-minutes per week.

Sleep Quality: Sleep quality was evaluated using the Pittsburgh Sleep Quality Index (PSQI), a widely used and validated questionnaire for assessing sleep patterns and disturbances [33]. The total PSQI score ranges from 0 to 21, with lower scores indicating better sleep quality.

Residential Context: Participants were classified according to their place of residence based on the population size and degree of urbanization, following the categorization proposed by the Atlas Estadístico de las Áreas Urbanas en España (Ministerio de Transportes, Movilidad y Agenda Urbana, 2024) [20]. This classification distinguishes between three main types of residential environments.

Large urban areas included municipalities with more than 50,000 inhabitants. Cities referred to small urban areas with a population between 20,000 and 50,000 inhabitants. Finally, municipalities were considered to be those towns or villages with fewer than 20,000 inhabitants.

This classification was used to capture potential differences in lifestyle behaviors, environmental characteristics, and access to health-related resources according to the degree of urbanization in which the participants lived.

### 2.4. Statistical Analyses

Normality of the data was evaluated using the Kolmogorov–Smirnov test, which is appropriate given the sample size (n = 571). Additionally, Q–Q plots and histogram visualizations of residuals were examined. Levene’s test was applied to assess the assumption of homogeneity of variances. For group comparisons, Student’s *t*-test was used when normal distribution was confirmed, whereas the Mann–Whitney U test was applied for non-normally distributed variables. Categorical variables were analyzed with the chi-square test, and differences between sexes were explored through post hoc analyses using adjusted residuals.

A linear regression analysis was conducted to estimate quality-of-life scores, with the model stratified by sex and adjusted for age, waist-to-height ratio, HDL and LDL cholesterol levels, adherence to the Mediterranean diet, sleep quality, physical activity level, and residential context. The KINDL score was included as the dependent variable. All covariates were standardized. Assumptions of linear regression were assessed, including normality of residuals (Q–Q plots and Kolmogorov–Smirnov test), homoscedasticity (visual inspection of residual plots), and absence of multicollinearity (variance inflation factor, VIF). Inferential *t*-tests and *p*-values were adjusted for heteroscedasticity. Post hoc tests with the Bonferroni correction were applied when a significant main effect was reported for a categorical predictor. Confidence intervals were estimated using bootstrapping.

The adequacy of the model was evaluated using the log-likelihood ratio test. A significance threshold of α < 0.05 was established for all analyses. Descriptive statistics are presented as means with standard deviations (SDs), medians with interquartile ranges (IQR: Q1–Q3), or frequencies, depending on the nature of each variable.

All statistical analyses were conducted using JAMOVI software for Mac (version 2.6.44, 2025) [34], incorporating the GAMLj module to perform generalized linear models [35].

## 3. Results

Statistically significant differences between sexes were reported for height (*p* = 0.043), waist-to-height ratio (*p* < 0.001), waist (*p* < 0.001), fat mass percentage (*p* < 0.001), muscle mass (*p* = 0.001), physical activity levels (*p* < 0.001), and HDL (*p* < 0.001) and LDL levels (*p* < 0.001). Descriptive statistics and group comparisons between boys and girls are presented in Table 1.

The full results of the multivariate analysis are presented in Table 2. A statistically significant effect of the Age variable on the self-reported quality of life of the participants was observed. The older the participants, the lower the participants’ self-reported quality of life (b = −1.07 95% CI [−1.61, −0.53], β = −0.15, t = −3.78, *p* < 0.001).

A statistically significant effect of the KIDMED variable on the self-reported quality of life of the participants was observed. Higher levels of adherence to the Mediterranean diet were associated with higher self-reported quality of life by participants (b = 0.72 95% CI [0.09, 1.37], β = 0.10, t = 2.29, *p* = 0.023).

A statistically significant effect of the PSQI variable on the self-reported quality of life of the participants was observed. Higher scores of PSQI (i.e., lower levels of sleep quality) were associated with lower self-reported quality of life by participants (b = −2.48 95% CI [−3.03, −1.93], β = −0.35, t = −8.67, *p* < 0.001).

**Figure 1:**
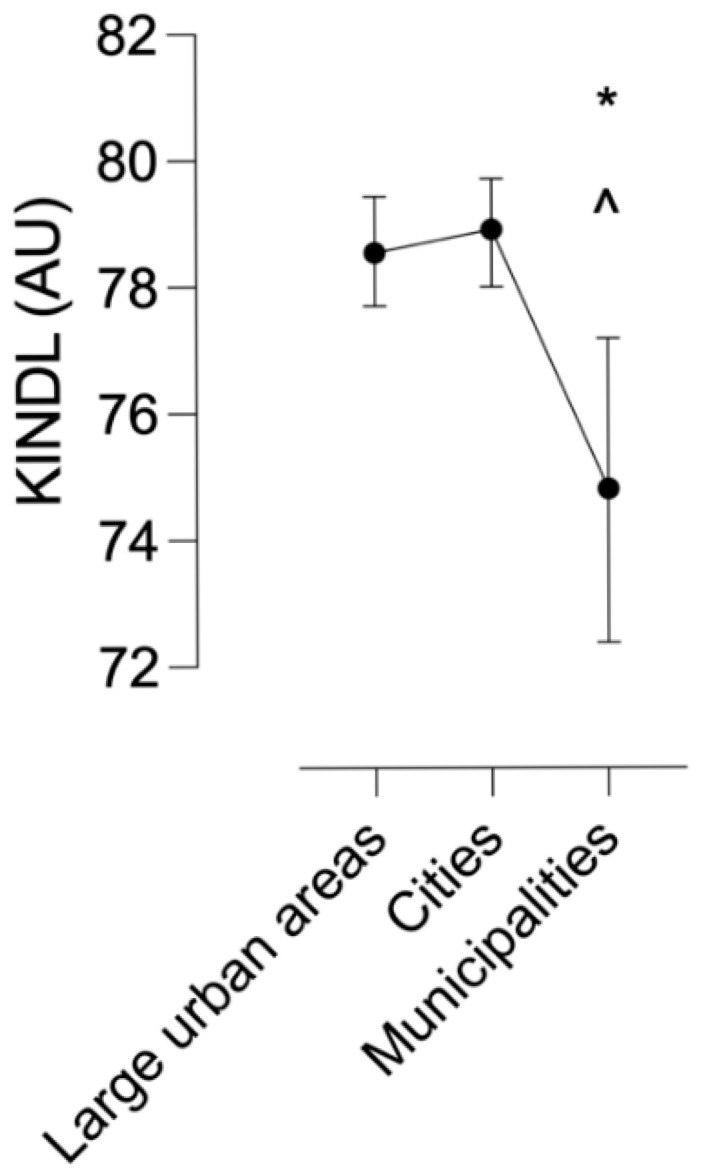
Self-reported quality of life scores by population. KINDL: Generic instrument for assessing health-related quality of life in children and adolescents aged 8 years and older, AU: Arbitrary units. * Significant difference compared to large urban areas (*p* < 0.05). ^ Significant difference compared to cities (*p* < 0.05).

A statistically significant effect of the Residential Context variable on the self-reported quality of life of the participants was observed (F = 4.61, *p* = 0.010). Higher levels of quality of life were reported in cities than in municipalities (MD = 4.10 ± 1.35, t = 3.03, *p* = 0.008, d = 0.65 95% [−0.83, −0.47]). Higher levels of quality of life were reported in large urban areas than in cities (MD = 3.73 ± 1.35, t = 2.76, *p* = 0.018, d = 0.59 95% [0.42, 0.77]). See Figure 1.

As shown in Figure 1, participants living in large urban areas and cities reported higher HRQoL scores compared to those residing in smaller municipalities. The decline in scores among children from municipalities was statistically significant, suggesting that population size and urban context may play a relevant role in shaping perceived quality of life.

## 4. Discussion

The present study aimed to analyze the associations between HRQoL and a set of demographic, anthropometric, biochemical, lifestyle, and environmental variables in a sample of school-aged children and adolescents. The findings revealed that age, adherence to the Mediterranean diet, sleep quality, and population type were significant predictors of HRQoL, whereas sex, WtH, lipid profile, and physical activity levels did not show statistically significant associations.

Consistent with previous research, a negative association was found between age and HRQoL, indicating that older participants reported lower levels of perceived quality of life [5,36]. This decline with age may reflect the increasing academic pressure, emotional challenges, and social demands that typically emerge during adolescence, factors that have been associated with a higher risk of mental health problems in this developmental stage [37]. Additionally, as children grow older, body image concerns tend to intensify, particularly during puberty, when physical changes may lead to greater dissatisfaction with one’s own appearance, negatively affecting emotional well-being and social relationships [38]. Another factor that may contribute to this decline is the progressive reduction of unstructured free time available for leisure and play. As academic demands increase, opportunities for spontaneous play, recreation, and peer interaction decrease, which may limit important sources of enjoyment and stress relief during childhood [39].

In contrast, sex did not emerge as a significant predictor of HRQoL in the present study, which contrasts with earlier literature reporting lower HRQoL in girls, particularly in emotional and psychological domains [5,40]. It is possible that this discrepancy is attributable to contextual or cultural factors specific to the studied population or to the relatively young mean age of the sample, where sex differences may not yet be strongly manifested.

Regarding anthropometric variables, the WtH was not significantly associated with HRQoL. While some studies have highlighted the negative impact of overweight and central adiposity on quality of life in children [18], the non-significant result observed here may suggest that, in relatively healthy and sportive school populations, anthropometric indicators exert less influence on subjective well-being compared to lifestyle factors.

Similarly, HDL-C and LDL-C were not related to HRQoL in this sample. Although alterations in lipid profile, particularly dyslipidemia, are well-established risk factors for cardiovascular disease from early ages [30], their direct influence on perceived well-being during childhood and adolescence appears to be limited. Previous research has shown that lipid alterations are present in European adolescents and are influenced by biological and anthropometric factors [41]. However, since dyslipidemia is usually asymptomatic during youth, its potential impact on quality of life is likely to be less perceptible compared to lifestyle behaviors that have an immediate effect on daily functioning, such as diet or sleep. Moreover, the active lifestyle commonly observed in sports school populations may contribute to maintaining cholesterol levels within healthy ranges, thereby reducing the likelihood of detecting differences in HRQoL related to lipid profile.

Importantly, adherence to the Mediterranean diet showed a positive and significant association with HRQoL. These findings align with previous evidence indicating that healthier dietary patterns are linked to better physical, emotional, and social well-being among children and adolescents [30,42]. This relationship may reflect not only the physiological benefits of a balanced diet but also psychosocial factors such as family eating routines or food-related satisfaction [31] (Serra-Majem et al., 2004).

Sleep quality emerged as one of the strongest predictors of HRQoL, with poorer sleep associated with significantly lower quality of life. This result is consistent with the literature highlighting sleep disturbances as a major determinant of psychological well-being, mood regulation, and cognitive functioning in pediatric populations [14,43]. Insufficient or poor-quality sleep has been linked to increased levels of stress, irritability, depressive symptoms, and difficulties in concentration and academic performance [44,45], all of which may ultimately influence subjective perceptions of quality of life.

The fact that sleep quality was one of the most relevant predictors in our model reinforces the idea that sleep is a fundamental pillar of health, often overlooked in pediatric interventions compared to other behaviors such as physical activity or diet. This result is particularly relevant given that recent studies have reported a worrying trend towards reduced sleep duration and increased sleep problems in school-aged children and adolescents, partly driven by excessive screen time, academic overload, and poor sleep hygiene habits [46]. In this context, promoting healthy sleep habits should be considered a key target for improving well-being in young populations, not only from a clinical perspective but also in terms of health promotion and prevention strategies at the school and community levels.

Although physical activity levels did not reach statistical significance in the regression model, the positive trend observed aligns with previous findings reporting beneficial effects of active lifestyles on HRQoL, particularly in physical and psychological domains [47]. One possible explanation for this non-significant association could be the relatively homogeneous physical activity levels across the sample, which may have reduced the variability needed to detect significant differences. This is especially plausible considering that participants were recruited from sports schools, where engagement in physical activity is likely to be systematically higher than in the general pediatric population. Furthermore, it is possible that the effect of physical activity on HRQoL operates indirectly, through its influence on other variables such as sleep quality, emotional well-being, or body image, which were included in the present model. Future studies including more heterogeneous samples in terms of physical activity levels might help to clarify the independent contribution of active lifestyles to perceived well-being in children and adolescents.

Another novel contribution of this study was the association found between residential context and HRQoL. Specifically, participants living in cities and large urban areas reported higher levels of HRQoL compared to those living in small municipalities. While some studies have reported better HRQoL in rural settings due to lower stress levels or closer social ties [48], other research has highlighted the advantages of urban environments, such as greater access to healthcare, sports facilities, and educational or recreational resources [49]. The present findings support the latter perspective, suggesting that the urban context may offer conditions that positively influence children’s well-being.

Several factors could explain this pattern. Urban environments may provide more opportunities for participation in structured leisure activities, organized sports, or social events, which can contribute to enhancing both physical and social dimensions of HRQoL [50]. In addition, the greater availability of specialized health and educational services in cities might facilitate early detection and management of health or emotional problems [51], ultimately improving children’s perception of their well-being. Conversely, smaller municipalities might offer fewer resources and opportunities for young people, potentially limiting their options for recreation, social interaction, or support services [51]. These differences highlight the importance of considering the local environment when designing public health interventions, ensuring that children and adolescents living in rural areas have equitable access to opportunities that foster their health and quality of life.

From a practical perspective, these findings highlight the importance of adopting a multifactorial approach when analyzing HRQoL in pediatric populations. Considering lifestyle factors (diet and sleep), sociodemographic variables (age and place of residence), and health indicators (anthropometry and lipid profile) within the same analytical framework allows for a more comprehensive understanding of the determinants of well-being. This integrative perspective is essential for the design of effective and targeted interventions aiming to promote health and quality of life among children and adolescents [52].

This study offers a comprehensive and integrated perspective on the determinants of health-related quality of life in youth by simultaneously considering lifestyle behaviors, anthropometric and biochemical markers, and residential context. Unlike prior research that has typically explored these factors in isolation, our findings highlight the combined influence of behavioral and environmental variables—particularly sleep quality and residential context—over traditional cardiometabolic indicators. These results emphasize the need for multidimensional frameworks in health promotion strategies targeting pediatric populations and reinforce the value of subjective well-being as a meaningful outcome in public health research.

Future research should consider the use of longitudinal designs to better understand causal relationships between lifestyle, biological, and environmental factors and HRQoL. Additionally, intervention studies targeting sleep quality, diet, and physical activity could help assess the effectiveness of behavioral strategies to enhance well-being in youth. Expanding the diversity of samples—geographically, socioeconomically, and culturally—would also be essential to improve the generalizability of findings and to identify population-specific determinants of quality of life.

The strengths of this study include the relatively large and diverse sample, the use of validated instruments for all variables, and the inclusion of multiple potential determinants of HRQoL analyzed simultaneously within a multifactorial model. This comprehensive approach enhances the understanding of the complex interplay between lifestyle, anthropometric, biochemical, and environmental factors in shaping quality of life among children and adolescents. Moreover, the use of robust and appropriate statistical methods, such as generalized linear models, contributed to the accuracy and reliability of the results, allowing for the adjustment of potential confounders and a more precise estimation of the independent effect of each variable on HRQoL.

However, some limitations should be acknowledged. First, the cross-sectional design limits the ability to establish causal relationships between the variables analyzed. Second, the reliance on self-reported measures, particularly for HRQoL, physical activity, and sleep quality, may introduce reporting bias, as responses could be influenced by social desirability or recall errors. In conclusion, this study provides new evidence on the complex interplay between lifestyle, biological, and environmental factors in shaping HRQoL among school-aged children and adolescents. Age, adherence to the Mediterranean diet, sleep quality, and population type emerged as significant predictors of HRQoL, underscoring the need for multidimensional health promotion strategies that address dietary habits, sleep hygiene, and equitable access to health resources across different living environments.

## 5. Conclusions

This study highlights key associations between lifestyle, biological, and contextual factors and health-related quality of life in youth. Sleep quality, adherence to the Mediterranean diet, and residential context emerged as the most relevant correlates, whereas biochemical markers such as HDL and LDL cholesterol showed weaker associations in this healthy and active sample.

These findings support the need to focus not only on individual health behaviors but also on environmental inequalities when designing strategies to promote well-being in children and adolescents. Future research should explore these relationships using longitudinal and intervention-based approaches to clarify causality and inform targeted public health actions.

By adopting a multidimensional perspective, this study contributes to a more integrative understanding of pediatric well-being and offers a practical framework for future research and health promotion initiatives.

## Figures and Tables

**Table 1:** Descriptive characteristics of the study population.

Variable	All (n = 571)	Girls (n = 254)	Boys (n = 358)	*p*-Values
Age (years) ^a^	11.64 ± 1.64	11.63 ± 1.65	11.65 ± 1.64	0.870
Height (m) ^a^	1.53 ± 0.12	1.52 ± 0.11	1.54 ± 0.13	**0.043**
Weight (kg) ^a^	46.87 ± 12.09	46.61 ± 11.37	47.05 ± 12.60	0.658
WtH (cm·m^−1^) ^a^	0.43 ± 0.05	0.42 ± 0.06	0.43 ± 0.05	** <0.001 **
Waist (cm) ^b^	65.80 ± 10.00	64.00 ± 9.13	66.50 ± 11.00	** <0.001 **
Fat mass (%) ^a^	23.25 ± 6.21	25.61 ± 5.01	21.40 ± 6.44	** <0.001 **
Muscle mass (kg) ^a^	33.96 ± 8.01	32.52 ± 6.87	35.08 ± 8.65	** 0.001 **
Tanner stage, I–V ^c^	101/192/163/116/20	34/73/74/56/9	67/119/89/60/11	0.145
IPAQ (MET-min·week^−1^) ^a^	2.99 ± 0.60	2.84 ± 0.56	3.09 ± 0.60	** <0.001 **
KIDMED (AU) ^a^	6.19 ± 2.44	6.34 ± 2.51	6.09 ± 2.38	0.229
PSQI, 0–21 (AU) ^b^	3.00 ± 2.00	3.00 ± 2.00	3.00 ± 2.00	0.871
HDL (mmMol·dL^−1^) ^a^	48.13 ± 15.10	52.04 ± 14.29	45.35 ± 15.06	** <0.001 **
LDL (mmMol·dL^−1^) ^a^	71.85 ± 25.32	76.51 ± 23.80	68.54 ± 25.87	** <0.001 **

Values in bold indicate statistically significant results (*p* < 0.05). Abbreviations: WtH: Waist-to-height ratio, Waist: Waist circumference, IPAQ: International Physical Activity Questionnaire, KIDMED: Adherence to the Mediterranean diet questionnaire, PSQI: Pittsburgh Sleep Quality Index, HDL: High-density lipoprotein, LDL: Low-density lipoprotein. ^a^ Data are presented as mean (SD), and differences between boys and girls were examined by analysis of variance. ^b^ Data are presented as median (IQR), and differences between boys and girls were examined by independent-samples Mann–Whitney U test. ^c^ Data are presented as frequency (%), and differences between boys and girls were examined by independent-samples chi-square test.

**Table 2:** The parameter Estimates (Coefficients) of the general linear model.

Effect	Estimate	SE	Lower 95% CI	Upper 95% CI	*β*	t	*p*
(Intercept)	77.42	0.50	76.45	78.35	−0.11	153.34	<0.001
Sex (B-G)	0.19	0.97	−1.67	2.10	0.03	0.19	0.847
Age (years)	−1.07	0.28	−1.61	−0.53	−0.15	−3.78	**<0.001**
WtH (cm·m^−1^)	−0.12	0.28	−0.64	0.43	−0.02	−0.44	0.661
HDL (mmMol·dL^−1^)	−0.31	0.38	−1.01	0.43	−0.04	−0.84	0.404
LDL (mmMol·dL^−1^)	0.25	0.36	−0.39	0.92	0.04	0.69	0.493
KIDMED (AU)	0.72	0.32	0.09	1.37	0.10	2.29	**0.023**
Large urban areas–Cities ^a^	−0.37	0.61	−1.50	0.73	−0.05	−0.60	0.546
Municipalities–Cities ^a^	−4.10	1.35	−6.78	−1.66	−0.58	−3.03	**0.003**
IPAQ (MET-min·week^−1^)	0.54	0.32	−0.04	1.19	0.08	1.69	0.093
PSQI, 0–21 (AU)	−2.48	0.29	−3.03	−1.93	−0.35	−8.67	**<0.001**
(B-G) × (Large urban areas–Cities ^a^)	−0.17	1.23	−2.51	2.22	−0.02	−0.14	0.893
(B-G) × (Municipalities–Cities ^a^)	3.87	2.72	−0.90	8.93	0.55	1.42	0.155

Values in bold indicate statistically significant results (*p* < 0.05). Abbreviations: B: Boys, G: Girls, WtH: Waist-to-height ratio, HDL: High-density lipoprotein, LDL: Low-density lipoprotein, KIDMED: Adherence to the Mediterranean diet questionnaire, IPAQ: International Physical Activity Questionnaire, PSQI: Pittsburgh Sleep Quality Index. Large urban areas: Municipalities with more than 50,000 inhabitants, Cities: Small urban areas with a population between 20,000 and 50,000 inhabitants, Municipalities: towns or villages with fewer than 20,000 inhabitants. ^a^ In this contrast Cities act as reference category.

## Data Availability

The data presented in this study are available on request from the corresponding author due to ethical restrictions related to the protection of sensitive information and privacy of underage participants, in accordance with institutional and data protection regulations.

## Abbreviations

The following abbreviations are used in this manuscript:

HRQoL	Health-related quality of life
WtH	Waist-to-height ratio
HDL	High-density lipoprotein
LDL	Low-density lipoprotein
